# Osteocyte Alterations Induce Osteoclastogenesis in an In Vitro Model of Gaucher Disease

**DOI:** 10.3390/ijms18010112

**Published:** 2017-01-13

**Authors:** Constanza Bondar, Maximiliano Ormazabal, Andrea Crivaro, Malena Ferreyra-Compagnucci, María Victoria Delpino, Paula Adriana Rozenfeld, Juan Marcos Mucci

**Affiliations:** 1 Instituto de Estudios Inmunológicos y Fisiopatológicos (IIFP), CONICET-UNLP, 1900 La Plata, Argentina; cmbondar@gmail.com (C.B.); maxi.ormazabal@gmail.com (M.O.); andrea.crivaro@gmail.com (A.C.); malenaferreyra@gmail.com (M.F.-C.); juanmarcosmucci@gmail.com (J.M.M.); 2 Instituto de Inmunología, Genética y Metabolismo (INIGEM), Hospital de Clínicas “José de San Martín”, Facultad de Medicina, CONICET-Universidad de Buenos Aires, Paraguay 2155, C1121ABG Buenos Aires, Argentina; victuare@gmail.com

**Keywords:** Gaucher disease, bone, osteocyte, osteoclast, apoptotic bodies

## Abstract

Gaucher disease (GD) is caused by mutations in the glucosylceramidase β (*GBA 1*) gene that confer a deficient level of activity of glucocerebrosidase (GCase). This deficiency leads to the accumulation of the glycolipid glucocerebroside in the lysosomes of cells, mainly in the monocyte/macrophage lineage. Its mildest form is Type I GD, characterized by non-neuronopathic involvement. Bone compromise is the most disabling aspect of the Gaucher disease. However, the pathophysiological aspects of skeletal alterations are not yet fully understood. The bone tissue homeostasis is maintained by a balance between resorption of old bone by osteoclasts and new bone formation by osteoblasts. A central player in this balance is the osteocyte as it controls both processes. We studied the involvement of osteocytes in an in vitro chemical model of Gaucher disease. The osteocyte cell line MLO-Y4 was exposed to conduritol-β-epoxide (CBE), an inhibitor of GCase, for a period of 7, 14 and 21 days. Conditioned media from CBE-treated osteocytes was found to induce osteoclast differentiation. GCase inhibition caused alterations in Cx43 expression and distribution pattern and an increase in osteocyte apoptosis. Osteoclast differentiation involved osteocyte apoptotic bodies, receptor activator of nuclear factor κ-B ligand (RANKL) and soluble factors. Thus, our results indicate that osteocytes may have a role to play in the bone pathophysiology of GD.

## 1. Introduction

Gaucher disease (GD) (Online Mendelian Inheritance in Man ID: 230800) is the most prevalent lysosomal disorder, caused by pathogenic mutations in the *GBA1* gene, leading to a deficient activity of the lysosomal enzyme glucocerebrosidase (GCase) (Enzyme Commission 3.2.1.45). This enzyme deficiency results in the accumulation of its substrate glucosylceramide, mainly in macrophages [1]. Phenotypically, patients may display three clinical forms. Type I is the most frequent form, it is chronic and non-neuropathic, and is characterized by visceromegalies, hematological alterations, and skeletal problems. Bone disease in Gaucher patients is a major matter of concern for physicians as it causes high morbidity and reduces quality of life. The main clinical manifestations of bone are acute and chronic pain, reduced bone density, osteonecrosis and osteolytic lesions [2]. Until now, the physiopathology of bone problems in Gaucher disease is not completely elucidated [3].

Bone is a mineralized connective tissue, which contains embedded osteocytes, and is covered by bone lining cells, osteoclasts, reversal cells and osteoblasts [4]. Furthermore, bone is a living organ in continuous remodeling. Bone remodeling is a highly complex process of resorption by osteoclasts and matrix formation by osteoblasts.

Osteoclasts are multinucleated cells that derive from fusion of cells of monocyte/macrophage lineage under the influence of various molecular mediators. One of the main factors influencing osteoclast generation is macrophage colony stimulating factor (M-CSF), which by binding to its receptor (colony stimulating factor 1 receptor) in precursors of osteoclasts, stimulates proliferation and inhibits apoptosis. Another important factor is receptor activator of nuclear factor κ-B ligand (RANKL), a protein expressed by stromal cells, osteoblasts and osteocytes. The binding of RANKL to RANK expressed in osteoclast precursors, induces osteoclastogenesis. The molecule called osteoprotegerin (OPG) is also participating in this process, as an inhibitor, by its interaction with RANKL [5].

Osteocytes are long-lived cells that comprise 90%–95% of the total bone cells. Osteocytes derive from osteoblasts and are located in the bone matrix forming the osteocyte lacunocanalicular system [6]. Cytoplasmic processes from different osteocytes, as well as from osteoblasts and bone lining cells, are connected by gap junctions. Interactions between osteocytes and the bone matrix are mediated by integrins [7]. Connexin-43 (Cx43) is a protein present in gap junctions; it mediates cell–cell coupling of adjacent osteocytes, and between osteocytes and bone surface cells [8]. Previous studies suggest that Cx43 hemichannels play a predominant role in maintaining osteocyte viability, which is essential for bone integrity and longevity. In fact, a decrease in Cx43 gap junction and hemichannel expression impairs osteocyte survival/function and leads to endocortical bone resorption by osteoclasts [9,10]. Osteocyte apoptosis acts as a chemotactic signal for osteoclasts in order to enhance bone resorption and engulf apoptotic bodies [11]. Moreover, a disruption in Cx43 mediated cell-to-cell communication between osteocytes may induce the release of local pro-osteoclastogenic cytokines [9]. Osteocyte-apoptotic bodies also have a potent osteoclastogenic activity, independently of osteoclastogenic factors [12]. Viable osteocytes nearby the dying osteocytes constitute the main source of RANKL, tumor necrosis factor α (TNF-α), interleukin 6 (IL6) and interleukin-1β (IL-1β) [13]. Therefore, osteocytes clearly participate in the regulation of osteoclastogenesis. 

Studies using different models of Gaucher disease have shown the involvement of osteoblasts in the bone pathophysiology of the disease. Reduced osteoblast proliferation and activity were found in mice and zebrafish models [14,15,16]. Therefore, bone alterations observed in Gaucher patients could be explained, at least partially, by changes in bone generating cells. On the other hand, our group and others have demonstrated that GCase deficiency is associated with increased osteoclastogenesis and bone resorption both in in vitro models and patients’ samples [16,17,18,19,20]. 

Taking into account that osteocytes play an important role in regulating osteoclastogenesis, we hypothesize that osteocyte biology may also be affected by GCase deficiency and are involved in bone alterations. Our aim was to evaluate the effect of GCase-deficient osteocytes on osteoclastogenesis, and we have proved that GCase deficiency in osteocytes increases the cellular apoptosis rate and induces osteoclastogenesis.

## 2. Results

### 2.1. Conditioned Media from CBE-Treated Osteocytes Induces BMM-Derived Osteoclastogenesis

**Figure 1:**
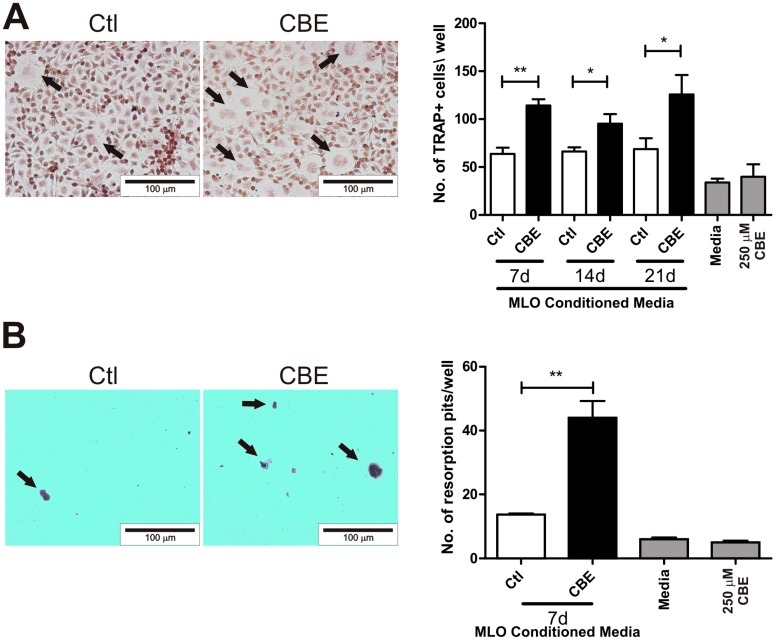
Induction of osteoclast differentiation by osteocyte conditioned media. MLO-Y4 cells were treated with conduritol-β-epoxide (CBE) for seven, 14 and 21 days (d), and conditioned media were harvested. Osteoclast precursors were exposed to conditioned media, and osteoclast generation was analyzed. Tartrate resistant acid phosphatase (TRAP) positive cells with ≥3 nuclei were counted (**A**); and the number of resorption lacunae were evaluated in osteoclast precursors exposed to conditioned media from osteocytes treated with CBE for seven days (**B**). Black arrows indicate osteoclast-like cells (**A**) or resorption lacunae (**B**), respectively. As control conditions, osteoclast precursors were treated with complete media (Media) or 250 µM of CBE. Data are expressed as mean ± SD (*n* = 5) and are representative of three independent experiments performed. * *p* < 0.05, ** *p* < 0.01 *t*-test.

Bone resorption is mediated mainly by osteoclasts, which originate from the fusion of cells from the monocyte-macrophage lineage [21]. Osteoclast maturation is mediated by RANKL, but, in some pathological situations, this can be induced or enhanced by proinflammatory signals [22,23,24]. MLO-Y4 cells were cultured in the presence of conduritol-β-epoxide (CBE) for seven, 14 and 21 days, and conditioned media were harvested and used in osteoclast differentiation assays. Osteoclastogenesis was evaluated using bone marrow-derived macrophages (BMM) stimulated with M-CSF and conditioned media from osteocytes. Osteoclast differentiation and activity were evaluated by the generation of multinucleated tartrate resistant acid phosphatase (TRAP) positive cells and dentine resorption, respectively. Conditioned media from CBE-treated osteocytes induced a higher number of osteoclast-like cells compared to untreated osteocytes at all time points tested (Figure 1A). Moreover, these cells presented resorptive activity determined by counting the number of resorption pits when treated with conditioned media from seven days of treatment (Figure 1B). BMM cells were cultured in the presence of complete medium or 250 µM CBE as controls, but no differences in osteoclastogenesis were observed compared to control conditioned media. These results indicate that GCase-deficient osteocytes generate factors that increase osteoclast differentiation.

### 2.2. GCase Deficiency Reduces the Expression of Integrin-β and Cx43 and Alters Cx43 Distribution Pattern 

**Figure 2:**
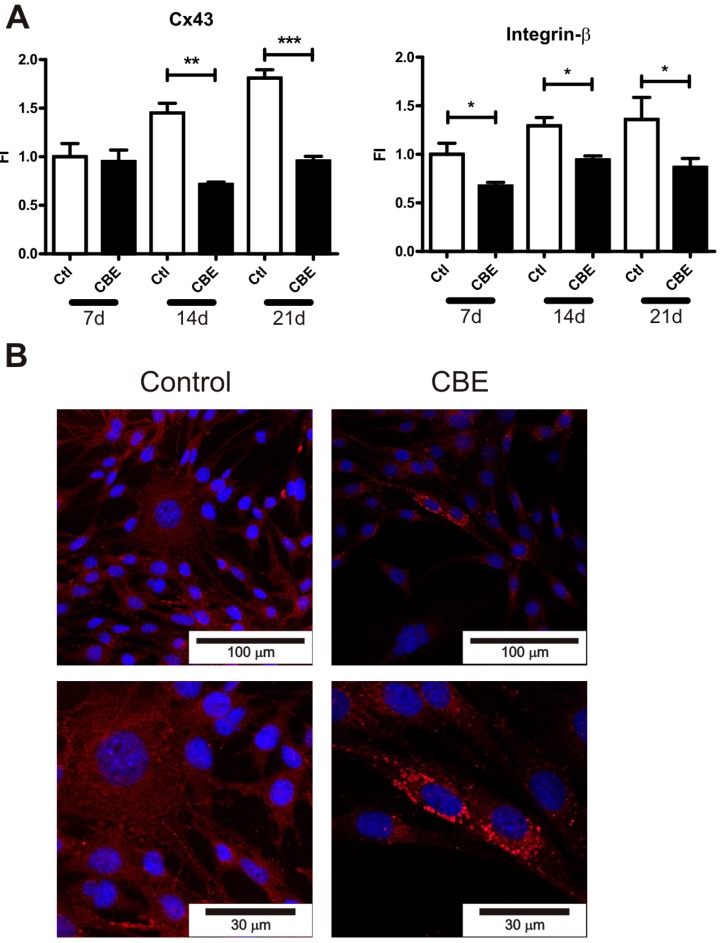

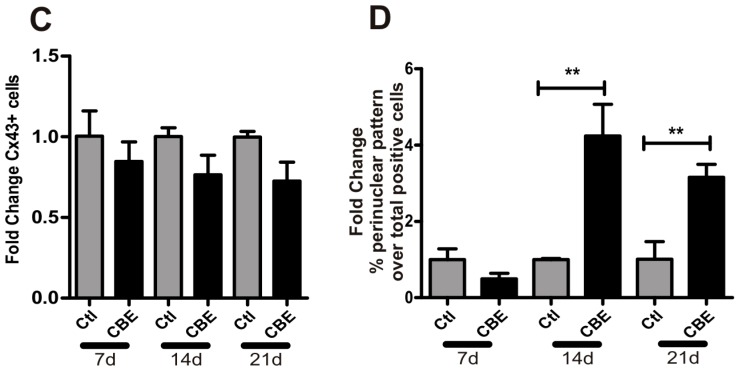
Connexin 43 expression and distribution are altered following CBE treatment. Relative expression of Cx43 and Integrin-β were assessed by qPCR in CBE-treated osteocytes at days (d) 7, 14 and 21 (**A**) Data were expressed as fold increase of gene expression (2^−Δ*C*t^) in the cells treated with CBE versus untreated cells (FI).; Cx43 presence was revealed by immunofluorescence in osteocytes treated with CBE for 7, 14 and 21 days. A representative image of each condition is shown using two different augmentations (**B**); the fold change of Cx-43 positive cells relative to the number of total cells (**C**) and the fold change in the percentage of cells presenting a perinuclear pattern relative to the total number of positive cells (**D**) were evaluated. Data are expressed as mean ± SD (*n* = 5) and are representative of three independent experiments performed. * *p* < 0.05, ** *p <* 0.01, *** *p* < 0.001, *t*-test.

Cx43 is the main gap junction protein in osteocytes. Together with integrins, Cx43 is involved in cellular communication and attachment to extracellular matrix processes required for osteocyte survival [8,9,10]. On the other hand, it has been shown that osteocyte apoptosis can induce the recruitment and differentiation of osteoclast precursors [25]. In this context, we evaluated the expression of several integrins and Cx43 as a possible mechanism leading to osteocyte cell death. The expression levels of CD44, E11/gp38, integrin-α, integrin-β and Cx43 were evaluated in CBE-treated osteocytes on days 7, 14 and 21. As shown in Figure 2A, Cx43 mRNA levels were lower on CBE-treated cells at the end of days 14 and 21, while integrin-β was downregulated at all time points tested. No differences were observed in the rest of the molecules analyzed.

To assess the protein levels of Cx43, immunofluorescence was carried out. The percentage of Cx-43 positive cells presented a trend towards a decrease in osteocytes treated with CBE in the 14 and 21 day periods (Figure 2C), while the Cx43 distribution pattern showed a significant difference, as it shifted from a widespread distribution in control cells to a perinuclear distribution in CBE-treated cells within the same time points (Figure 2B,D).

### 2.3. CBE Treatment Induces Osteocyte Apoptosis

Osteocytes from mice lacking Cx43 exhibit increased apoptosis [9]. As CBE-treatment reduced the expression and altered the pattern distribution of Cx43, we aimed to investigate the possible induction of osteocyte apoptosis in GCase-deficie mnt osteocytes. For this purpose, osteocytes were cultured in the presence of CBE for 7 and 14 days, and apoptosis was evaluated by terminal deoxynucleotidyl transferase dUTP nick end labeling (TUNEL) and Annexin V-phycoerythrin (PE)/propidium iodide (PI) staining.

**Figure 3:**
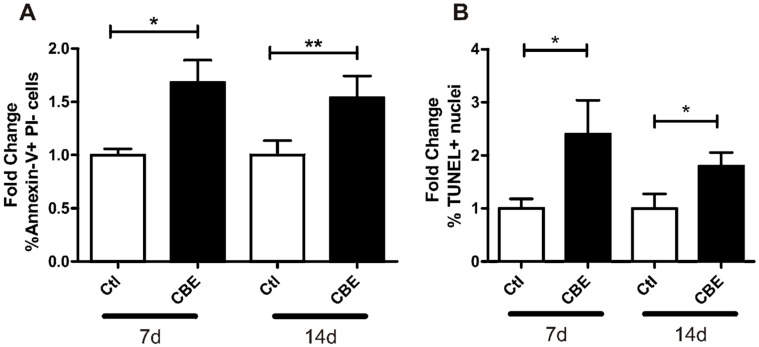

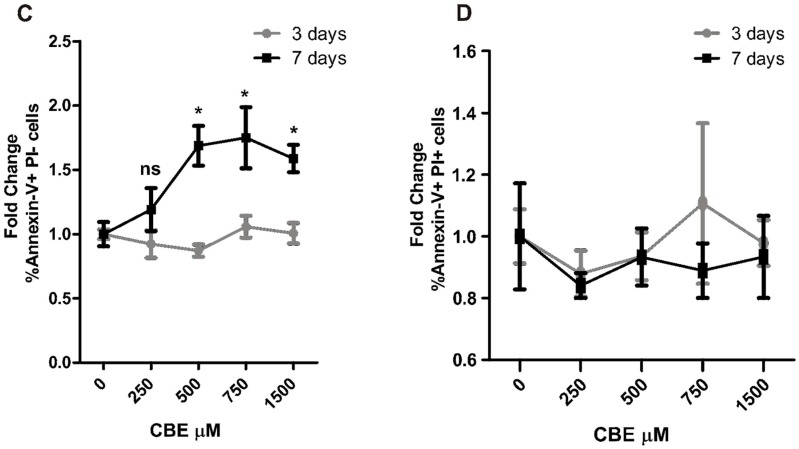
GCase deficiency induces osteocyte apoptosis. Osteocytes were treated for 7 and 14 days with CBE, and apoptosis was evaluated by Annexin-V^+^ (**A**) and terminal deoxynucleotidyl transferase dUTP nick end labeling (TUNEL) (**B**). The fold change between CBE and control cells for the percentage of Annexin-V^+^ propidium iodide (PI)^−^ osteocytes (**A**) and the percentage of TUNEL^+^ nuclei (**B**) are shown. Results are presented as the fold change for CBE treatment against control conditions. Data are expressed as mean ± SD (*n* = 5) and are representative of three independent experiments performed. * *p* < 0.05, ** *p* < 0.01 *t*-test. CBE toxicity was evaluated by Annexin-V staining in osteocytes treated with different concentrations of CBE for three and 7 days. The fold change between CBE and control cells for the percentage of Annexin-V^+^PI^−^ (**C**) and Annexin-V^+^PI^+^ (**D**) osteocytes is shown. Data are expressed as mean ± SD (*n* = 5) and are representative of three independent experiments performed. * *p* < 0.05 one-way ANOVA. ns, non-significant.

We found evidence of a significant increase in the percentage of apoptotic cells treated with CBE for 7 and 14 days (Figure 3A,B). This indicates that GCase deficiency in osteocytes leads to an increase in the apoptotic state in osteocytes.

To discriminate if the increased apoptosis was a consequence of the accumulation of glucosylceramide or if CBE could have by itself a toxic effect, MLO-Y4 cells were treated with increasing concentrations of CBE for three and 7 days. A 250 µM concentration of CBE inhibited GCase activity to 13.8% ± 0.2%, which was not sufficient to generate substrate accumulation (Figure S1); on the other hand, CBE concentrations of 500 µM or more inhibited enzyme activity to less than 4% and generated substrate accumulation (Figure S1). At each time and concentration tested, Annexin-V staining was performed. As shown in Figure 3C, the presence of CBE for three days did not induce apoptosis. However, the seven-day treatment induced apoptosis only in concentrations of 500 µM and higher, with no differences among them. Moreover, the number of necrotic cells (Annexin-V^+^PI^+^) remained unchanged for all treatments and time points tested (Figure 3D). These results rule out any toxic effects of CBE and show that an accumulation of glucosylceramide for seven days is needed to trigger the apoptotic events.

### 2.4. Osteoclastogenesis Induction by Conditioned Media from CBE-Treated Osteocytes Involves Apoptotic Bodies, RANKL and Soluble Factors

**Figure 4:**
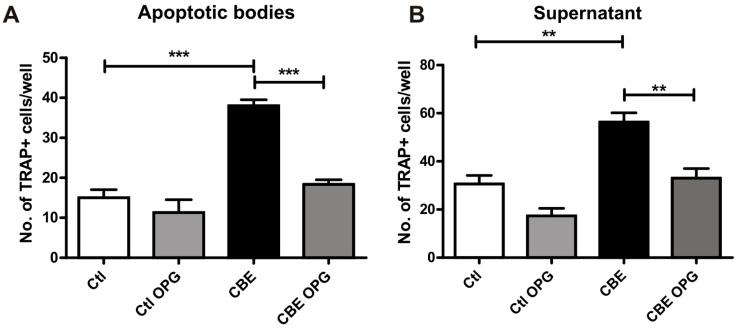
Osteoclast differentiation by conditioned media involves apoptotic bodies and receptor activator of nuclear factor κ-B ligand (RANKL). MLO-Y4 cells were treated with CBE for seven days and conditioned media were harvested and centrifuged to obtain apoptotic body and supernatant fractions. Osteoclast differentiation assays were performed in the presence of the fraction containing the apoptotic bodies (**A**) or the supernatant fraction (**B**). Experiments were carried out in the presence or absence of recombinant osteoprotegerin (OPG) to evaluate RANKL involvement. + cells with ≥3 nuclei were counted. Data are expressed as mean ± SD (*n* = 5) and are representative of three independent experiments performed. ** *p* < 0.01 *** *p* < 0.001 one-way ANOVA.

Osteocytes undergoing apoptosis produce apoptotic bodies that can recruit osteoclast precursors and induce osteoclast differentiation [12,25]. Given the increase in osteocyte apoptosis induced by CBE treatment (Figure 3) and in osteoclast differentiation induced by conditioned media from CBE-treated osteocytes (Figure 1), we decided to investigate the involvement of apoptotic bodies and RANKL in the osteoclast differentiation process. To evaluate these effects, osteocytes were treated with CBE for seven days, while conditioned media were harvested and subjected to high speed centrifugation to isolate the apoptotic bodies. The apoptotic bodies and the supernatant were then used in BMM osteoclast differentiation assays in the presence or absence of recombinant OPG to evaluate RANKL involvement. Our results showed that both the apoptotic bodies and the supernatant from CBE-treated osteocytes induced higher levels of osteoclast-like cells when compared to osteoclast precursors treated with supernatants from untreated osteocytes (Figure 4A,B). When OPG was added to the apoptotic bodies from CBE-treated osteocytes, the number of osteoclast-like cells decreased to those observed in control (Figure 4A). This indicates that RANKL on the surface of apoptotic bodies is the main inducer of osteoclast differentiation. On the other hand, the addition of OPG to the supernatant led to a decrease in osteoclast-like cells in the CBE conditioned media treated cells and a decreasing trend in the control conditioned media treated cells (Figure 4B). This result indicates that soluble RANKL present in control and CBE supernatants is the main factor involved in osteoclastogenesis.

**Figure 5:**
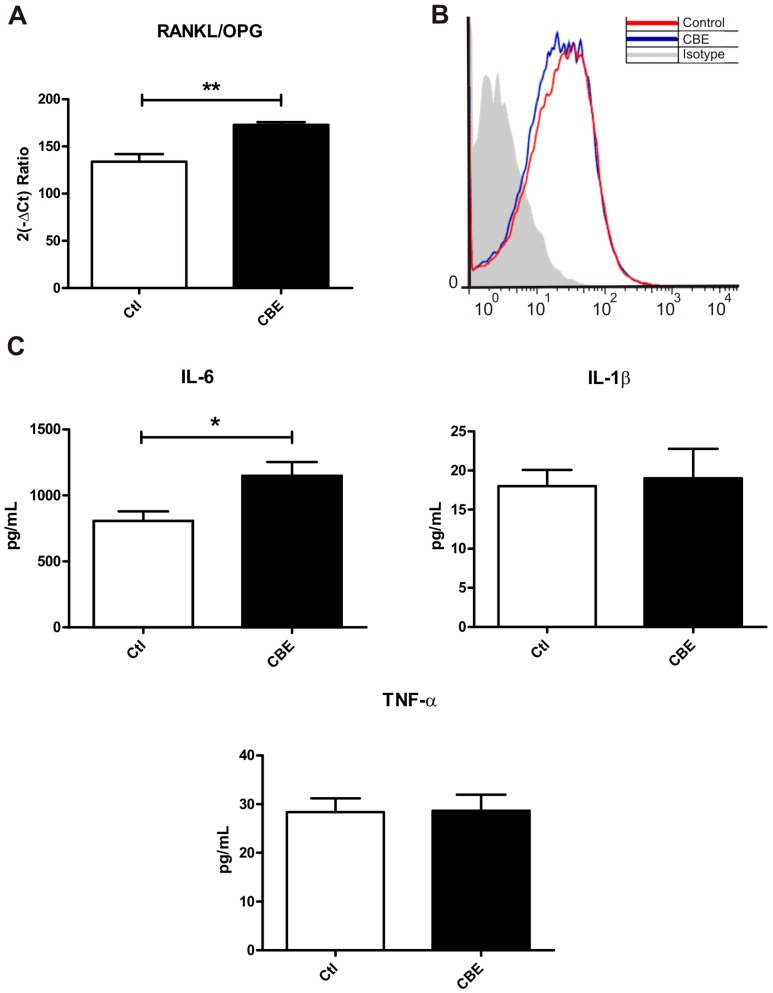
RANKL/OPG ratio and interleukin 6 (IL-6) are increased by CBE treatment. MLO-Y4 cells were treated with CBE for 7 days. Cells and conditioned media were harvested. Cells were used for qPCR analysis and the ratio between the mRNA levels for RANKL and OPG was evaluated (**A**); the conditioned media was centrifuged to obtain the fraction containing the apoptotic bodies and the supernatant fraction. Surface levels of RANKL in the fraction containing the apoptotic bodies were analyzed by flow cytometry (**B**); a representative histogram of each condition, as derived from the three independent experiments conducted, is shown. IL-6 levels were evaluated by capture ELISA in the supernatant fraction (**C**). Data are expressed as mean ± SD (*n* = 5) and are representative of three independent experiment performed. * *p* < 0.05, ** *p* < 0.01 *t*-test.

Therefore, it was decided to evaluate the levels of RANKL, OPG and cytokines involved in the osteoclast differentiation process by CBE-treated osteocytes. For this purpose, osteocytes were treated for seven days with CBE, and the expression levels of mRNA for RANKL and OPG were evaluated. Even though no statistically significant differences were observed in the expression levels of both molecules, a significant increase in the RANKL/OPG ratio was found in CBE-treated osteocytes compared to control cells (Figure 5A). This increased ratio may contribute to the induction of osteoclastogenesis.

Fluorescence associated cell sorting (FACS) analysis were then performed to evaluate membrane-bound RANKL levels in the fraction containing the apoptotic bodies. As shown in Figure 5B, no differences were present in RANKL levels in this fraction derived from CBE or control cells. Thus, differences in the oseoclastogenesis induced by the apoptotic bodies are likely to be caused by higher levels of apoptotic bodies from CBE-treated cells vis-à-vis untreated cells, rather than to a difference in surface RANKL levels in individual apoptotic bodies. 

Other soluble factors involved in the induction of osteoclastogenesis were analyzed as well (Figure 4B). The pro-osteoclastogenic cytokines IL-1β, TNF-α and IL-6 were assessed in the supernatant by ELISA. While IL-1β and TNF-α levels were not altered by CBE treatment, IL-6 release from CBE-treated osteocytes was significantly increased (Figure 5C). This increment in IL-6 levels may contribute to the osteoclast differentiation process induced by supernatants.

## 3. Discussion

Bone involvement is one of the most debilitating features of Gaucher disease [2,26]. There are currently two main theories to explain the pathophysiological mechanisms involved in this feature. One of them is based on the results obtained from an animal model, which found evidence of a decrease in bone generation but no alterations in bone degradation [14]. In this model, the malfunction of osteoblasts was shown to be related to a decrease in the proliferation and viability of these cells caused by the toxic effects of circulating bioactive lipids [14,27]. In line with this concept, a zebrafish model of Gaucher disease showed impaired osteoblast differentiation and reduced bone mineralization associated with a defective canonical Wnt signaling pathway [15].

The second theory, involving the participation of osteoclasts in the bone pathophysiology of Gaucher disease, has been proved in in vitro models and cultures of patient’s peripheral blood mononuclear cells (PBMC). Models using murine or human cells have shown that glucocerebrosidase deficiency leads to the release of proinflammatory mediators, which cause the differentiation of active osteoclasts. Among these molecules, IL-6 and TNF-α were proposed as the main mediators of the process [16,17,19]. Remarkably, patients presented a higher number of osteoclast precursors in circulation. In addition, the culture of patients’ PBMCs in the presence of osteoclastogenic factors gave rise to more mature osteoclasts than control PBMCs, in a process involving TNF-α and RANKL [18,20].

The osteocyte is the most abundant cell type in bone, but its role in bone dynamics was not described until recently [13,28,29,30]. As osteocytes have been proved to be involved in the regulation of bone remodeling, we aimed to study the involvement of these cells in the alterations of bone homeostasis in GD. To this end, we used the MLO-Y4 osteocyte-like cell line as an in vitro model of osteocytes. This cell line presents a number of limitations for the study of osteocytes and its correlation to in vivo conditions. MLO-Y4 cells lack the expression of osteocyte factors such as sclerostin [31]. Osteocytes are buried in the bone matrix, and the three-dimensional environment in which they are immersed is essential to their morphology and function [32]. Despite these limitations, the use of this cell line as an in vitro model has been shown to be a powerful tool to study osteocyte physiology and the relationship between osteocytes and other bone cell types, including osteoclasts [31,33,34,35,36]. To evaluate the role of GCase-deficient osteocytes in osteoclastogenesis, we performed osteoclast differentiation assays in the presence of conditioned media from osteocyte cultures. Such assays revealed that conditioned media from CBE-treated osteocytes induced higher osteoclast differentiation and resorptive activity when compared to conditioned media from untreated cells. This increase in osteoclast differentiation and activity is likely to lead to higher bone resorption in GD.

To further understand the mechanisms involved in osteoclast differentiation mediated by conditioned media, osteocyte apoptosis was analyzed. This process has been shown to induce the recruitment of osteoclast precursors as well as osteoclast differentiation both in vitro and in vivo [12,25]. To this end, we examined the mRNA levels of several proteins involved in osteocyte survival. Connexin-43, the most abundant protein of the connexin family expressed in bone cells, mediates cell-to-cell communication as well as cell–matrix interactions [8]. It has been shown that Cx43 deficiency in osteocytes leads to increased apoptosis in these cells [9]. In addition, integrins also influence the fate of osteocytes by regulating cell proliferation and apoptosis [37]. We found that both Cx43 and integrin-β expression were lower in CBE-treated osteocytes. Moreover, Cx43 protein distribution pattern was highly altered, and this shift from a widespread to a perinuclear pattern could lead to a decrease in Cx43 hemichannels in the membrane, which, in turn, could decrease cell–cell and cell–matrix communication [9,10,38]. As these changes may lead to a higher cell death in CBE-treated osteocytes, Annexin-V and TUNEL staining were performed, demonstrating that GCase deficiency induces osteocyte apoptosis.

Apoptotic bodies derived from osteocytes are capable of initiating osteoclast differentiation in vivo and in vitro [12,25]. Here, we have shown that osteoclast differentiation is induced more significantly by apoptotic bodies from CBE-treated osteocytes than by apoptotic bodies from untreated cells. Moreover, RANKL has been proved to be involved in this process, while surface RANKL levels in apoptotic bodies have been found to show no differences between control and treated cells. Thus, differences in osteoclastogenesis induction are likely to be caused by the increased apoptosis observed in CBE-treated osteocytes, and, consequently, by the higher number of apoptotic bodies. It has been proposed that apoptotic bodies are a signal from dying osteocytes to increase the levels of RANKL in neighboring viable osteocytes. This might explain the increase in the RANKL/OPG ratio observed in CBE-treated osteocytes. As a two-step centrifugation protocol was used to isolate the apoptotic bodies, we cannot rule out the presence of any microvesicles and necrotic cells in this fraction [39]. As necrotic osteocytes do not induce osteoclast differentiation [12], further studies will be required to evaluate both the presence of microvesicles in the fraction containing the apoptotic bodies as well as their involvement in the osteoclast differentiation process.

Our results indicate that the supernatant from CBE-treated osteocytes induces higher osteoclast differentiation and that soluble RANKL is not likely to be the only molecule involved. Several soluble molecules are capable of inducing osteoclastogenesis, either RANKL-dependent or RANKL-independent. Among them, IL-6 and TNF-α can give rise to mature osteoclasts in the absence of RANKL and have been shown to be upregulated in Gaucher disease models [23,40,41,42,43]. Both molecules were evaluated in the soluble fraction of CBE and control cells. Although soluble TNF-α levels were not different between the two fractions, IL-6 levels were higher in the CBE soluble fraction. These differences with other models of the disease are most probably due to the specific cell type studied, as other studies focused mainly on fibroblasts and brain cells. The increase in IL-6 might contribute to an increase in osteoclast differentiation induced by the soluble fraction derived from GCase-deficient cells.

## 4. Materials and Methods

### 4.1. Animals

The University of La Plata provided 6- to 8-week-old female C57Bl/6 mice, which were housed in groups of five and kept under controlled temperature (22 °C) and artificial light conditions during a 12-h cycle period. Mice were provided with sterile food and water ad libitum.

All experimental protocols in this study were conducted strictly in accordance with international ethical standards for animal experimentation (Declaration of Helsinki and its amendments, Protocol on Animal Welfare under Amsterdam Treaty and the Guide for the Care and Use of Laboratory Animals approved by the National Institutes of Health, Bethesda, MD, USA). The protocols in this study were approved by CICUAL (Comité Institucional para el Cuidado y Uso de Animales de Laboratorio de la Facultad de Ciencias Exactas de la Universidad Nacional de La Plata) (protocol 001-17-16/, 13SEP2016, La Plata, Buenos Aires).

### 4.2. Cell Culture and Apoptotic Body Isolation 

The murine osteocyte cell line MLO-Y4 was cultured in α-minimum essential medium (α-MEM) supplemented with 5% FBS, 5% bovine calf serum (Gibco-BRL, Carlsbad, CA, USA), 100 U of penicillin per ml and 100 μg/mL of streptomycin, at 37 °C in a 5% CO_2_ atm for 7, 14 and 21 days in the presence or absence of CBE 500 µM (Matreya, State College, PA, USA). For all cultures, cells were placed on collagen (Gibco-BRL, Carlsbad, CA, USA) coated bottles or multiwell plates. The effect of CBE on glucocerebrosidase activity was tested, demonstrating the inhibition of enzyme activity to 3.9% ± 0.05% and accumulation of glucocerebroside by means of thin layer chromatography, as previously described [17]. The conditioned media were harvested three times and evaluated by centrifugation at 500× *g* for 30 min.

The apoptotic bodies and the supernatant studied were obtained following a two-step centrifugation protocol. At the indicated time points, the culture supernatant was harvested and centrifuged at 500× *g* for 30 min to remove cells and large debris. This medium was then centrifuged at 30,000× *g* for 30 min in order to separate the supernatant and the pellet, which was resuspended in 150 µL of serum free α-MEM to obtain the apoptotic bodies.

To study the effect of CBE treatment on osteocyte apoptosis, MLO-Y4 cells were treated with 500 µM of CBE for 7 and 14 days. Apoptosis levels were evaluated by Annexin-V and TUNEL staining.

To evaluate the toxicity of CBE, cells were treated with 250, 500, 750 and 1500 µM for 3 or 7 days and apoptosis was studied by Annexin-V staining.

### 4.3. Osteoclast Formation Assay

BMM from C57Bl/6 mice were plated at 5 × 10^5^ cells/500 µL and cultured at 37 °C in 5% CO_2_ atm in α-MEM supplemented with 2 mM l-glutamine, 10% fetal bovine serum (FBS) (Gibco-BRL, Carlsbad, CA, USA), 100 U of penicillin per mL and 100 µg of streptomycin per mL (complete medium) and 30 ng/mL of recombinant murine macrophage colony stimulating factor (M-CSF) (ImmunoTools, Friesoythe, Germany) for 72 h. Adherent cells were used for osteoclast differentiation assays. Cultures were performed in a complete medium supplemented with M-CSF for 7 days, replacing the media every 48 h. In the assays involving conditioned media and soluble fraction, each was added in a 1:1 ratio. In the experiments involving the fraction containing the apoptotic bodies, 30 µL of this fraction were added to evaluate osteoclastogenesis. Recombinant OPG (R&D Biosciences, Minneapolis, MN, USA) was used at 50 ng/mL to evaluate the involvement of RANKL. As a negative control, cultures received a complete medium and 250 or 500 µM of CBE supplemented with M-CSF. Osteoclasts were identified by tartrate-resistant acid phosphatase (TRAP; Sigma Aldrich, St. Louis, MO, USA) and hematoxylin stainings. Osteoclasts were defined as TRAP-positive multinucleated (more than 3 nuclei) cells, and their number was determined by counting at the microscopic level. 

### 4.4. Pit Formation Assay 

BMM from C57Bl/6 mice were plated at 2 × 10^4^ cells/0.25 mL/well on dentine disks (Osteo Assay Multiwell Strips, Corning Life Science, Lowell, CA, USA) in 96-well culture dishes in a complete medium and 30 ng/mL of recombinant murine macrophage colony stimulating factor (M-CSF) (ImmunoTools) for 72 h. Adherent cells were used for resorption pit assays. Adherent cells were cultured in conditioned media and a complete medium in a 1:1 ratio containing M-CSF (30 ng/mL) for 6 days. Media and all reagents were replaced every day to avoid the acidification of the medium. After the cells were cultured, dentine disks were washed with 1M NH_4_OH to remove adherent cells. After rinsing with water, dentine disks were stained in 1% Toluidine Blue/1% sodium borate and visualized by light microscopy (Sigma Aldrich, St. Louis, MO, USA) to determine the number of resorption lacunae.

### 4.5. Flow Cytometry

The fraction containing the apoptotic bodies was resuspended in phosphate buffer saline (PBS) containing 10% of normal mouse serum, and either anti-RANKL antibody (Biolegend, San Diego, CA, USA, cat 510005) or an isotype control (Biolegend cat 400507) was added. After 20 min of incubation at 4 °C, samples were analyzed on a FACSCalibur flow cytometer (BD Pharminigen, San Diego, CA, USA).

### 4.6. Annexin-V Staining

MLO-Y4 cells were harvested by treatment with TRyple solution (Gibco-BRL, Carlsbad, CA, USA) and washed with fresh medium. Cells were centrifuged and resuspended in 50 µL of binding buffer (HEPES 10 mM, NaCl 140 mM, CaCL_2_ 2.5 mM, pH = 7.40); then, 1 μL of Annexin V-APC (BD Pharminigen, San Diego, CA, USA) and 1 μL of propidium iodide (1 mg/mL) (Sigma) were added. After a 30-min incubation at room temperature, samples were analyzed by flow cytometry in a FACScalibur (Becton Dickinson, Franklin Lakes, NJ, USA).

### 4.7. Real Time PCR

Total RNA was isolated from MLO-Y4 cells by means of a total RNA isolation system (GE Healthcare, Piscataway, NJ, USA) following the manufacturer's protocols. The isolated total RNA samples were then reverse transcribed using random hexamers and reverse transcriptase (Invitrogen, Carlsbad, CA, USA). Real-time quantitative PCR (qPCR) was performed using SYBR GreenER PCR Master Mix (Invitrogen) in an iQ-Cycler equipment (Bio-Rad, Hercules, CA, USA). The sequence-specific primers were designed using PerlPrimer software (Sourceforge, San Diego, CA, USA). A mouse HPRT gene was used as an internal control. The comparative threshold (*C*_t_) method was used for data analysis. Data were expressed as fold increase of gene expression (2^−Δ*C*t^) in the cells treated with CBE versus untreated cells (FI).

### 4.8. Immunofluorescence Microscopy 

MLO-Y4 cells were grown in chamber slides as previously described and fixed in 4% paraformaldehyde for 10 min at room temperature. Cells were permeabilized with 0.3% Triton X-100 in PBS for 10 min and blocked with 5% goat serum for 30 min. For Cx43 staining, cells were incubated with a 1:100 dilution of mouse anti-Cx43 antibody (Thermo Fisher Scientific, Boston, MA, USA, cat 13-8300) and then with a 1:300 dilution of Alexa Fluor488 Fab´fragment of goat anti-mouse IgG (H + L) (Invitrogen, Carlsbad, CA, USA, cat A11020). Nuclei were stained with propidium iodide 1 µg/mL for 15 min (Sigma Aldrich, St. Louis, MO, USA, cat P4170). Samples were mounted using Fluorescent Mounting Medium (DakoCytomation, Glostrup, Denmark, cat S3023) and visualized in a TCS SP5 Leica confocal microscope (Leica Microsystems, Wetzlar, Germany). Images were taken using the Leica LAS AF software (Leica Microsystems, Wetzlar, Germany).

The primary antibody was incubated overnight at 4 °C, and washes between incubations were performed with 0.1% Tween-20 in PBS. Secondary antibodies were incubated for 1 h at room temperature.

Total count of nuclei was performed using the ImageJ software (National Institutes of Health, Bethesda, MD, USA). Positive cells were counted manually. Results were expressed as percentages.

### 4.9. TUNEL

Cells were grown in chamber slides and fixed in 4% paraformaldehyde. TUNEL imaging assays were conducted according to the manufacturer’s instructions (Dead End Fluorometric TUNEL system; Promega, Madison, WI, USA). Nuclei were stained with 4′,6-diamidino-2-phenylindole (DAPI) and mounted using Fluorescent Mounting Medium. Samples were visualized using a Nikon Eclipse Ti fluorescence microscope (Nikon, Sendai, Japan) as previously described. Total count of nuclei was performed using the ImageJ software. Positive cells were counted manually. Results were expressed as percentages.

### 4.10. Cytokine Measurement

IL-6, IL-1β and TNF-α levels in the supernatant fraction were quantified by capture ELISA (BD Pharminigen, San Diego, CA, USA) following the manufacturer’s instructions

### 4.11. Statistical Analysis 

Statistical analyses were performed using GraphPad Prism 5.0 software (Graphpad Software, La Jolla, CA, USA) applying *t*-test or one-way ANOVA, followed by post hoc Tukey Test. Data are expressed as mean ± SD (*n* = 5) and are representative of three independent experiments performed.

## 5. Conclusions

To conclude, our results showed that GCase deficiency in osteocytes leads to an increase in osteoclast differentiation through a mechanism involving osteocyte apoptosis and soluble molecules. This process may certainly contribute to bone pathogenesis in Gaucher disease.

## Supplementary Materials

Supplementary materials can be found at www.mdpi.com/1422-0067/18/1/112/s1.

## Abbreviations

GD	Gaucher disease
GCase	Glucocerebrosidase
CBE	Conduritol-β-epoxide
Cx-43	Connexin 43
RANKL	Receptor activator of nuclear factor κ-B ligand
RANK	Receptor activator of nuclear factor κ-B
M-CSF	Macrophage colony stimulating factor
TNF-α	Tumor necrosis factor-α
IL-6	Interleukin-6
IL-1β	Interleukin-1β
BMM	Bone marrow derived macrophage
TRAP	Tartrate resistant acid phosphatase
PI	Propidium iodide
OPG	Osteoprotegerin
FACS	Fluorescence associated cell sorting
TUNEL	Terminal deoxynucleotidyl transferase dUTP nick end labeling
