# Supplementary material for: Osteocyte Alterations Induce Osteoclastogenesis in an In Vitro Model of Gaucher Disease
Source: Int J Mol Sci. 2017 Jan 13;18(1):112. doi: 10.3390/ijms18010112 (PMC5297746; doi:10.3390/ijms18010112)
Supplement: Supplementary file 1 [file ijms-18-00112-s001.pdf]

Supplementary Materials: Osteocyte Alterations Induce Osteoclastogenesis in an In Vitro Model of Gaucher Disease

Constanza Bondar, Maximiliano Ormazabal, Andrea Crivaro, Malena Ferreyra-Compagnucci, María Victoria Delpino, Paula Adriana Rozenfeld and Juan Marcos Mucci

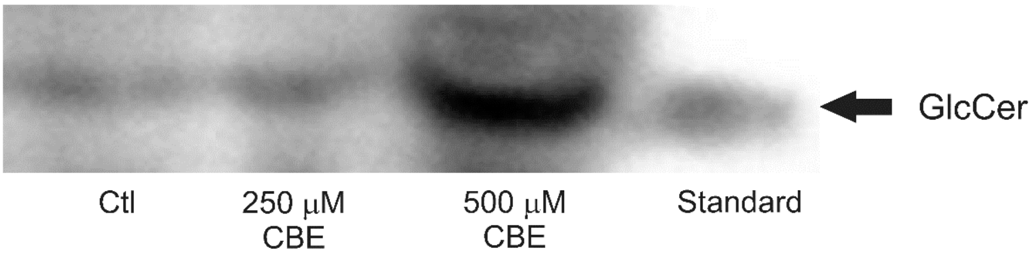

Figure S1. MLO-Y4 cells were treated with 0, 250 or 500 µM conduritol-β-epoxide (CBE) for 3 days and glucosylceramide levels were evaluated by thin layer chromatography (TLC) together with a standard. Black arrow indicates GlcCer. A picture is shown representative of three independent experiments performed.
